# An Ounce of Prevention from a Ton of Tomatoes

**DOI:** 10.1289/ehp.113-a178

**Published:** 2005-03

**Authors:** Cynthia Washam

Tomato processors enhancing the health of consumers with their nutrient-rich tomato extract soon may enhance the health of their communities as they adopt a new, green technique for producing the precious substance. A team of researchers from the University of Florida (UF) has developed an extraction method using supercritical carbon dioxide (CO_2_), which may provide a cleaner way to get more extract from more tomatoes.

Companies that produce tomato extract and pure lycopene (the carotenoid that gives tomatoes their red color) typically use chemical solvents to draw the compounds from tomatoes. One of the most commonly used solvents is ethyl acetate. Although nontoxic, ethyl acetate is highly flammable and must be removed from the extract through distillation.

“The advantage of supercritical extraction is that we use carbon dioxide,” says Murat Balaban, a professor of food engineering at the UF Institute of Food and Agricultural Sciences and leader of the research team. “Neither the remaining tomato nor the extract need to be cleaned of solvent as in the case of ethyl acetate, because carbon dioxide . . . is easily separated from them.” Supercritical extraction does not produce CO_2_, which is associated with global warming; it only uses it.

“This is another wonderful example of how people are using green chemistry to shift from traditional technology,” says Paul Anastas, director of the Green Chemistry Institute in Washington, D.C. “Nothing is as innocuous as carbon dioxide.”

## A Tomato a Day

Lycopene supplements became the darling of health-conscious Americans a decade ago, when studies began to link high tomato consumption with a reduced risk of several types of chronic disease. Some studies associated high tomato consumption with a reduced risk of heart disease. Others found evidence that tomatoes help protect against cancer of the prostate, digestive tract, cervix, breast, and lung. In the 6 March 2002 issue of the *Journal of the National Cancer Institute*, Harvard researcher Edward Giovannucci and colleagues reported that men who ate 2–4 servings of tomato sauce per week had a 35% reduced risk of prostate cancer, the second leading cause of cancer death in American men.

Until a year ago, many scientists believed the protection came solely from lycopene, which is the most prevalent carotenoid in the serum of people following a Western diet. Like beta-carotene, lutein, and other carotenoids, lycopene is an antioxidant. Antioxidants neutralize free radicals, errant molecules produced as by-products of cell metabolism that accumulate with age. Free radicals are implicated in cancer, arteriosclerosis, macular degeneration, and other degenerative diseases.

A more recent study suggests protection doesn’t come from lycopene alone, but from a mixture of carotenoids occurring naturally in tomatoes. A team of researchers from the University of Illinois at Urbana–Champaign and The Ohio State University reported that discovery in the 5 November 2003 issue of the *Journal of the National Cancer Institute*. The researchers fed lycopene to one group of rats with prostate cancer and powdered tomatoes to another. Rats treated with lycopene had the same death rate as controls, whereas those fed tomato powder had a 26% lower risk of prostate cancer death. In late 2004, a team of Northwestern University researchers launched a new study to further investigate lycopene’s potential as a prostate chemopreventive.

Still, for those too rushed to eat their tomatoes, supplements are an easy alternative, and men eager to ensure they got enough of the antioxidant to ward off prostate cancer have embraced lycopene supplements. Sales of lycopene in both 2001 and 2002 were $3 million, and approximately $4 million in 2003, according to Patrick Rey, research director of the *Nutrition Business Journal*. (Rey had no sales figures for tomato extract.)

## Red Fruit, Green Chemistry

The technology Balaban and his colleagues studied has been used for years in processing other edibles. Several major coffee and tea companies use supercritical CO_2_ extraction to remove caffeine. Breweries treat hops with the same technology to extract flavor and aroma. It is also used to freeze-dry some vegetables and remove fat from animal products such as powdered eggs. The pharmaceutical industry also uses supercritical CO_2_ extraction to remove medicinal compounds from herbs. No commercial tomato processors have used supercritical gas extraction, although Balaban expects that to change as news of his results spreads.

The small-scale experiments the UF researchers have been conducting begin with chopped, partly dried tomatoes. The pieces are placed in a closed chamber with controlled temperature and pressure. CO_2_ gas is pumped into the vessel and put under high pressure. Intense pressure causes the CO_2_ to become dense and behave like a liquid solvent. This liquefied, or supercritical, gas extracts carotenoids from the tomatoes much like hot water extracts flavonoids from tea. The addition of a small amount of ethanol to the CO_2_ enhances the extraction.

When the process is finished, the pressure is released and the CO_2_ returns to a gaseous state, leaving a concentrated mixture of several carotenoids, including lycopene. Removing pure lycopene from the mixture requires the additional step of chromatography. Balaban says the CO_2_ used in the process can be recaptured and used again, although the UF team has not done so.

Researchers at other universities have produced tomato extract with supercritical CO_2_, Balaban says, but none could match his team’s yields. He attributes his success to extensive studies (which have not yet been written up for publication) using various temperatures, pressures, and ethanol concentrations. The highest yields came from a pressure of 5,000 pounds per square inch, a temperature of 55°C, and a solvent that’s 90% CO_2_ and 10% ethanol. “It’s a good extraction,” Balaban says. “What we’re getting is extremely dark red.”

That’s no surprise to Val Krukonis, president of Phasex Corporation in Lawrence, Massachusetts. Phasex performs supercritical CO_2_ extraction for several medical and pharmaceutical companies. Krukonis says this type of extraction often produces a greater yield than extraction with solvents. Some manufacturers also choose supercritical CO_2_ extraction over solvent-based extraction because it can result in better color and taste.

## Relief for Farmers

The only reason, it seems, that not every manufacturer of coffee, tea, beer, or drugs has adopted supercritical CO_2_ extraction is its cost. The equipment used for supercritical CO_2_ extraction costs considerably more than equipment used for traditional solvent-based extractions. Operating costs also are higher because of the high pressure required.

“In general, it’s more expensive,” confirms Uy Nguyen, vice president of manufacturing for U.S. Nutraceuticals in Eustis, Florida. “Because of the cost, we apply the technology mainly to high-value products.”

Arguments over cost, though, don’t fully apply in the extract market the UF team has in mind. They developed their technology to help farmers who watch helplessly every year as a sizeable share of their crop goes to waste.

Florida tomatoes are cultivated to be sold fresh to consumers, not—like those grown in some other states—to commercial producers of soup, sauce, and other processed tomato products. So “eye appeal” is critical. To minimize bruising, Florida tomatoes are picked when they’re mature but still green. Ethylene gas treatment in the packinghouse jump-starts the ripening process that gives tomatoes their blush. Up to 20% of the Florida tomatoes that are picked are discarded at the packinghouse for cuts, bruises, or odd shapes.

Farmers also see the market price plummet during the late-season harvest. Tomato fields in the Sunshine State are typically picked three or more times, about five to seven days apart, as the fruit reaches maturity. Prices are highest for the first pickings, but often dip as the market becomes saturated. When prices are too low to justify the cost of harvest labor, farmers stop picking, no matter how much fruit is left on the vine. Every year, they abandon at least 10% of the crop.

“We harvest as long as the market will support it,” says John VanSickle, director of the UF International Agricultural Trade Policy Center. “We’ve had times when the market was so bad the field was abandoned after one picking.”

Farmers accustomed to losing a share of their annual crop would welcome even small profits on tomatoes that otherwise would go to waste, Balaban contends. “Our objective was to bring something to the tomato growers in Florida,” he says. “If we can use an otherwise useless material to do that, we will be contributing to their well-being.”

Balaban believes a company using supercritical CO_2_ extraction could keep its costs lower by buying unwanted tomatoes—those rejected at the plant and picked after the market for fresh tomatoes bottoms out. In comparison, growers who produce tomatoes specifically for lycopene production demand a higher price because it’s the only market for their crop. Israeli-based LycoRed, for example, holds the patent on a specially developed lycopene-rich hybrid tomato, which it licenses to farmers.

In a study not connected with Balaban’s, UF horticulture professor Steven Sargent found that more than 30% of the green tomatoes culled at packinghouses could be ripened with ethylene gas, increasing still more the number that could be used to make extract (those that remain green don’t have enough lycopene to be used in extract production). Culls from specialty tomatoes, such as grape, cherry, and Roma types, could also be used, says Sargent.

## Tomatoes of Tomorrow

Supercritical CO_2_ extraction may be costly today, but proponents of green technology see it becoming more economical tomorrow if more companies adopt the technology as they come under governmental pressure to reduce their use of hazardous materials. That demand, in turn, could spur more green innovations and help lower costs. “Companies using organic solvents face a tremendous number of regulations,” Anastas says. “Going beyond compliance is an excellent economic strategy.”

Krukonis agrees. “It’s not yet cost-effective,” he says of supercritical extraction of tomatoes, “but that doesn’t mean it won’t be in the future.”

Balaban would most like to see the technology used to produce extract that contains the full spectrum of tomato carotenoids. Pharmaceutical manufacturers pay $5,100 for a kilogram of extract, according to medical market analysts RAK Associates. Another potential market Balaban sees for his technology is producing pure lycopene for researchers. Most lycopene extracted for nutritional supplements is not pure enough for scientists, he explains. He knows of only one company that sells research-grade lycopene: St. Louis–based Sigma-Aldrich charges $106.50 for one milligram.

Whether his technology is used to produce lycopene for researchers or extract for consumers, Balaban sees a rosy future. “I think the demand is going to go up,” he says. “I’d like to see [a company using this technology] start small and grow.”

## Figures and Tables

**Figure:**
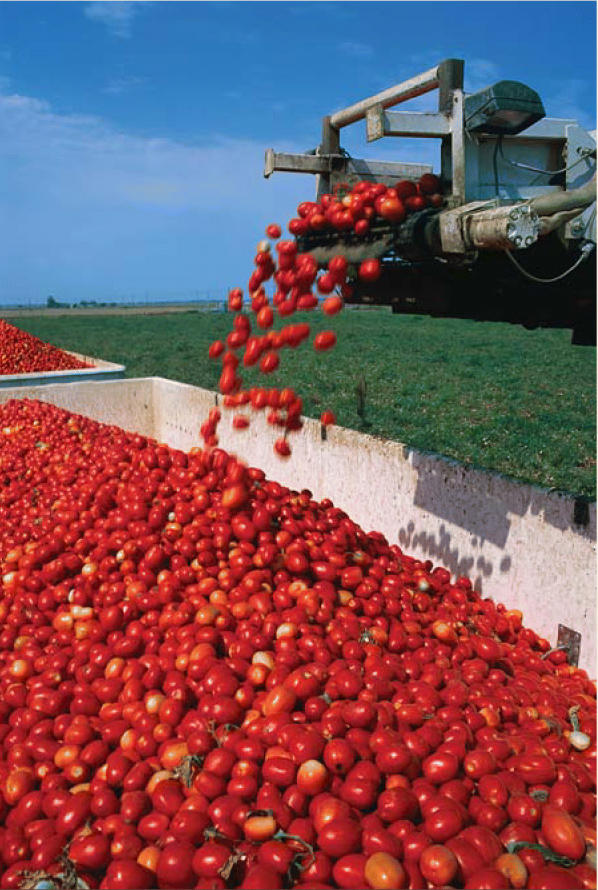
**Ripe with possibilities.** New techniques allow for cleaner and more effective extraction of health-protective substances from tomatoes.

**Figure:**
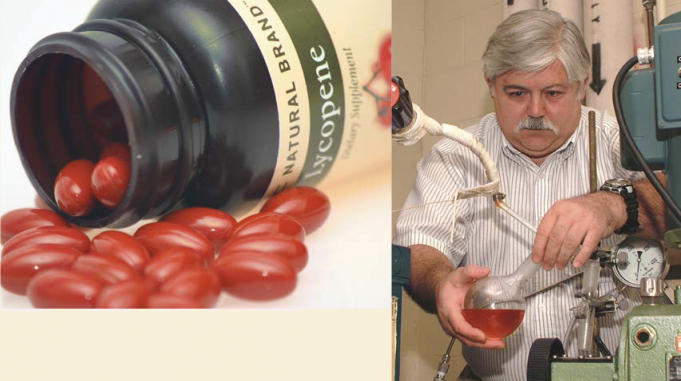
**Tomato tonic.** Murat Balaban (right) demonstrates the technology he’s developed to extract lycopene using supercritical CO_2_. Lycopene (above) is a hot-selling nutritional supplement among men who wish to prevent prostate cancer.
